# Spectrum of neuroimaging findings in COVID-19

**DOI:** 10.1259/bjr.20200812

**Published:** 2020-12-01

**Authors:** Ahmed H El Beltagi, Surjith Vattoth, Mohamed Abdelhady, Islam Ahmed, Yahya Paksoy, Mohamed Abou Kamar, Hussam Alsoub, Muna Almaslamani, Abdul Latif Alkhal, Ahmed Own, Ahmed Elsotouhy

**Affiliations:** 1 Neuroradiology Department, Neuroscience Institute, Hamad Medical Corporation, Doha, Qatar; 2 Clinical Imaging, Weill Cornell Medicine University - Qatar, Doha, Qatar; 3 University of Arkansas for Medical Sciences, Little Rock, AR, USA; 4 Infectious and Communicable Diseases Department, Hamad Medical Corporation, Doha, Qatar

## Abstract

An outbreak of corona virus disease 2019 (COVID-19) began in China in December 2019, and rapidly spread to become a worldwide pandemic. Neurological complications encountered in hospitalized patients include acute arterial ischemic cerebrovascular stroke, cerebral venous thrombosis, critical illness-associated cerebral microbleeds, hypertensive hemorrhagic posterior reversible encephalopathy, meningoencephalitis/flare up of infections, flare up of multiple sclerosis, acute disseminated encephalomyelitis**,** cerebral hemodynamic/hypoxic changes such as watershed ischemic changes and hypoxic ischemic encephalopathy, and spine manifestations of Guillain Barre syndrome and viral myelitis. The purpose of our study is to illustrate the different neuroimaging features in critically ill hospitalized COVID-19 positive patients in the State of Qatar.

## Introduction

An outbreak of coronavirus disease 2019 (COVID-19) began in Wuhan, China, in December 2019, and has rapidly spread around the world, and the COVID-19 severe acute respiratory syndrome corona virus-2 (SARS-CoV-2) was declared by the World Health Organization (WHO) as a pandemic on March 11, 2020. As of June 28, 2020, 10,238,287 people have tested COVID19 positive globally, with 504 078 reported deaths. In Qatar, 94 413 persons were proved COVID19 positive by the same date (3625 cases per million), with a total of 110 deaths (www.worldometers.info/coronavirus/)^1^

COVID-19 mostly affects the respiratory system, ranging from mild flulike symptoms, fever, cough, and/or dyspnea, to severe pneumonia, acute respiratory distress syndrome (ARDS), and respiratory failure. Extra respiratory multisystemic involvement has also been increasingly recognized, with cardiac injury, renal failure, septic shock and multiorgan failure.^2^

Li et al^3^ recently described the neurotropic and neuroinvasive potential of COVID-19. The neurological complications encountered in hospitalized patients include acute arterial ischemic cerebrovascular stroke, cerebral venous thrombosis (CVT), critical illness-associated cerebral microbleeds, hypertensive hemorrhagic posterior reversible encephalopathy (PRES), meningoencephalitis/flare up of infections, flare up of multiple sclerosis, hemodynamic changes such as watershed hypoxic ischemic changes and hypoxic ischemic encephalopathy, and spine manifestations of Guillain Barre syndrome and viral myelitis.

neurological complications encountered in hospitalized patients include acute arterial ischemic cerebrovascular stroke, cerebral venous thrombosis (CVT), critical illness-associated cerebral microbleeds, hypertensive hemorrhagic posterior reversible encephalopathy (PRES), meningoencephalitis/flare up of infections, flare up of multiple sclerosis, hemodynamic changes such as watershed hypoxic ischemic changes and hypoxic ischemic encephalopathy, and spine manifestations of Guillain Barre syndrome and viral myelitis.

The purpose of our study is to illustrate the different neuroimaging features in critically ill hospitalized COVID-19 positive patients in the State of Qatar.

### Acute ischemic stroke

The risk of developing acute ischemic stroke in patients with COVID-19 infection is more in those with advanced pneumonia and multiple organ dysfunctions. All aspects of the coagulation cascade are affected in severe viral infections such as H1N1-SARS, Ebola, Herpes Zoster, and others.And the fibrin D-dimer levels are 12-fold higher in patients with COVID-19 infection who developed stroke indicating a hypercoagulable state.^4^

**Figure 1.**
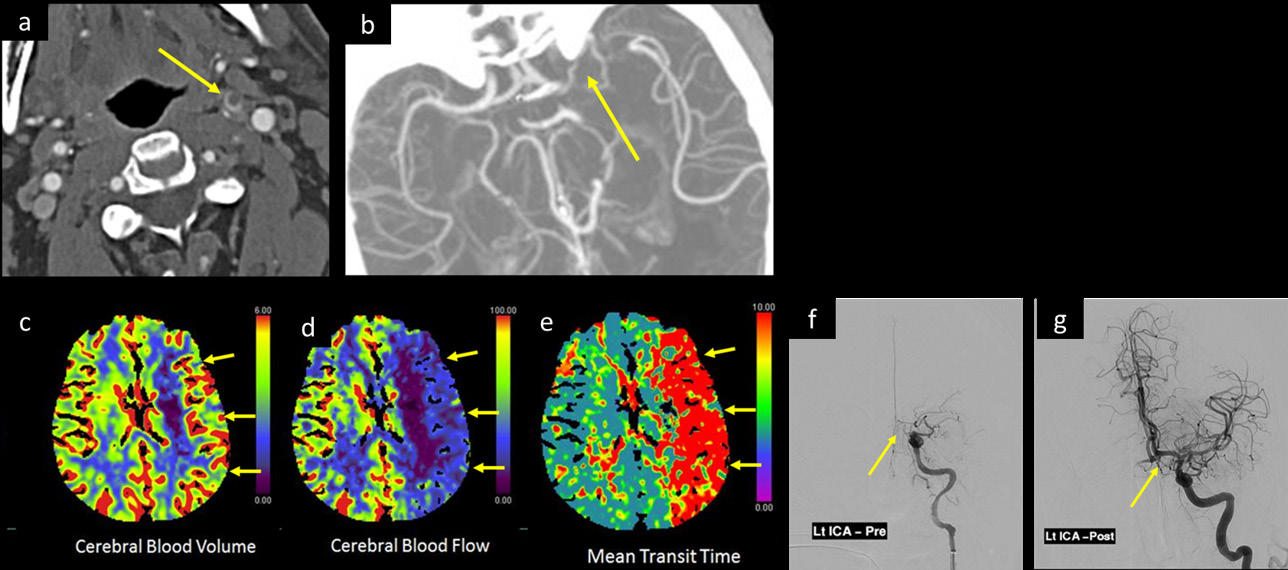
A 30-year-old male was tested positive for COVID-19 with marked deterioration of respiratory symptoms, suddenly developed aphasia and right-side-weakness with a low GCS. CTA of the head and neck, axial images at (a) suprahyoid neck and (b) at Sylvain fissure levels, show a floating thrombus within the cervical left ICA with intracranial extension occluding the left ICA terminus and M1 segment of left MCA (arrow in a and b). CTP of the brain post-processed (c) CBV image, (d) CBF, and (e) MTT color map images display large area of mismatched defect, with large perfusion defect along the left MCA territory showing decreased CBF, prolonged MTT, and compensated CBV (short arrows in c, d, and e respectively). Catheter angiography (f) before and (g) after successful thrombectomy with aspiration of the clot from left ICA show recanalization of left MCA and ACA. Unfortunately, few days later, the left ICA reoccluded. CBF, cerebral blood flow; CBV, cerebral bloodvolume; CTA, CT angiogram; CTP, CT perfusion; GCS, Glasgow coma scale; ICA, internalcarotid artery; MTT, mean transit time.

**Figure 2.**
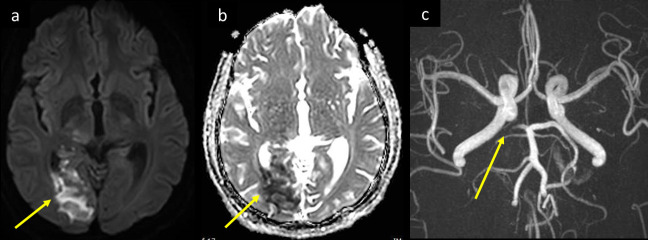
A 58-year-old male diabetic with fever and mild respiratory symptoms was tested positive for COVID-19. He developed confusion, and headache increasing in severity over the past 2 days, associated with dizziness, blurring of vision, and left homonymous hemianopia. MRI (a) axial DWI b 1000, and (b) ADC map shows bright signal and corresponding low signal respectively along the right occipital lobe (arrow in a and b). Intracranial 3D MIP MRA (c) shows thrombotic occlusion of the P2 segment of right posterior cerebral artery (arrow in c). ADC, apparent diffusion coefficient; DWI, diffusion-weighted image; MIP, maximum intensity projection; MRA,MR angiography.

**Figure 3.**
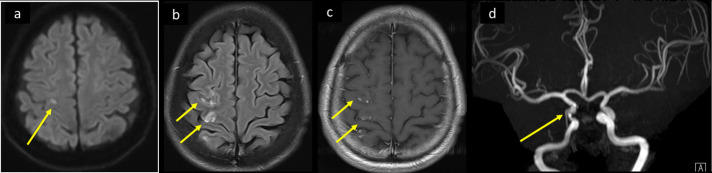
A 47-year-old male who was a chronic smoker, and had DM, HTN presented to the ED, with history of two episodes of weakness of the left side of the body that lasted for 1 h and totally resolved. He had fever and mild respiratory symptoms and tested positive for COVID-19. Axial MRI brain images at supraventricular level, (a) DWI b 1000, (b) T2-FLAIR and (c) *T*_1_WI post i.v. contrast show precentral tiny lacunar diffusion restriction with bright signal on DWI-b 1000 (arrow in a), pre- and post-central cortical and subcortical areas of bright signal on FLAIR *T*_2_WI, and gyral post-contrast enhancement (arrows in b, c respectively) consistent with acute to evolving subacute ischemic changes. This correlates with the resolved episodes of TIAs. (d) Intracranial MIP- 3D-MRA shows significant narrowing with mural irregularity of cavernous segment of right ICA (arrow in d) which probably along with COVID-19 increased thrombogenicity led to more distal small vessel emboli. DM, diabetes mellitus; ED,emergency department; FLAIR, fluid attenuated inversion recovery; HTN, hypertension;ICA, internal carotid artery; MIP, maximum intensity projection; MRA, MRangiography; TIA, transient ischemic attack.

**Figure 4.**
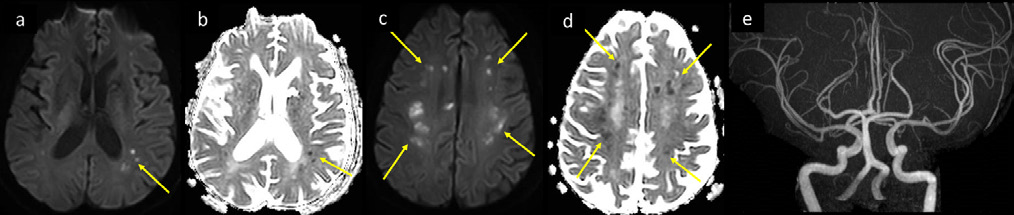
CNS vasculitis like changes in a 56-year-old male patient with COVID-19 severe ARDS. He was intubated with stabilization of pulmonary function and oxygen saturation, blood pressure was controlled, and coagulation panel was normalized. He remained drowsy and non-arousable. MRI axial DWI and ADC, (a, b) at the ventricular level, and (c, d) at supraventricular level showing left frontoparietal periventricular and multilobar bilateral deep subcortical recent lacunar infracts with diffusion restriction with punctate and nodular foci of bright DWI and corresponding low signal on ADC (arrows in a–d). The intracranial MRA (f) showed normal appearance of arteries. ADC, apparent diffusion coefficient; ARDS, acute respiratory distress syndrome; CNS, central nervous system; DWI, diffusion-weighted image; MRA,MR angiography.

As part of the host defense mechanism to limit spread of pathogens, tissue factor (TF) appears to be a major activator of the coagulation cascade during viral infection, and its expression is increased in endothelial cells infected with the virus. However, excessive activation of the coagulation cascade has a deleterious effect with increased risk of acute cerebrovascular stroke and cerebral venous thrombosis.^5^ Acute ischemic stroke in our case series were seen to involve large, (Figure 1) medium-sized (Figure 2) or small arteries (Figure 3). Moreover, a diffuse central nervous system vasculitis like pattern was also encountered in some cases (Figure 4). Small vessel microangiopathy has been related to propensity of COVID- 19 to infect endothelial cells of different vascular beds, the CoV spike glycoprotein binds to the angiotensin converting enzyme 2 (ACE2) receptor, The expression of the ACE2 receptor in neurons and cerebral endothelial cells indicates a high level of invasiveness for the SARSCoV-2 in comparison with other coronaviruses. Such endothelial mechanism of injury (endotheliitis) with histologic evidence of COVID-19-induced vasculitis has also been reported in several other organs including the heart, lung, liver, kidney, and skin.^6–8^

In a recent single center study of 221 hospitalized patients with COVID-19 infection, acute ischemic stroke occurred in 11 (5%); (5 large artery disease, 3 small artery disease and 3 cardioembolic), cerebral venous sinus thrombosis in 1 (0.5%), and cerebral hemorrhage in 1 (0.5%), with some patients having transient ischemic attack (TIA) as their initial presentation.^9^

### Cerebral venous thrombosis

**Figure 5.**
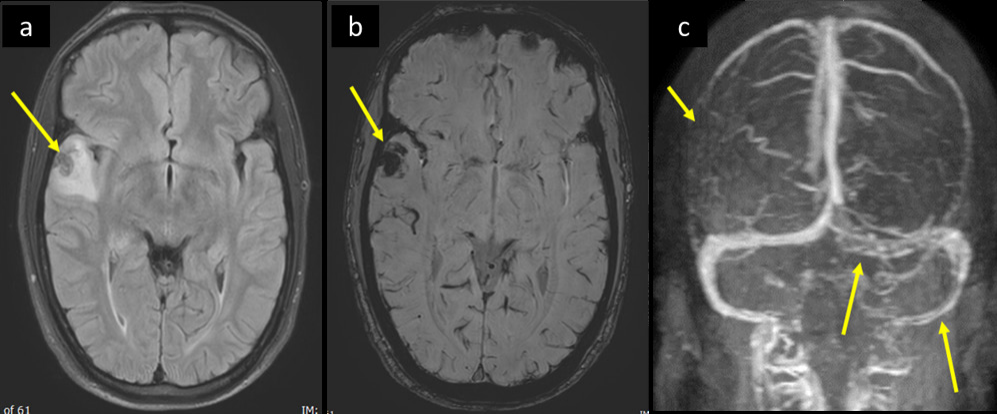
A 30-year-old male patient presented with recent episodes of tonic clonic seizures associated with post-ictal confusion. He had no fever, respiratory symptoms, or neck stiffness. He tested COVID positive by PCR. MRI brain, (a) axial FLAIR *T*_2_WI and (b) axial SWI shows right temporal intra-axial cortical and subcortical area of low signal on FLAIR *T*_2_WI with surrounding parenchymal bright signal edema, and blooming hypointensity in SWI images (arrow in a and b respectively). There was no diffusion restriction (not shown). (c) Source image of post-contrast MR venography shows filling defect in the torcula and left transverse sinus extending to the sigmoid sinus (long arrows in c), with abrupt cut-off of right superior anastomotic vein (short arrow in c). FLAIR, fluid attenuatedinversion recovery; PCR, polymerase chain reaction; SWI, susceptibility-weightedimaging.

As mentioned above, severe viral infections result in hypercoagulable state and increased thrombogenicity which can also precipitate cerebral venous thrombosis (CVT) (Figure 5).

The viral infection itself or increased inflammatory cells result in activation of the endothelial cells which produce procoagulant cytokines, that in turn induce the expression of prothrombotic endothelial cell proteins with shift of the endothelial cell surface from thromboresistance to a prothrombotic. It also affects primary hemostasis, with thrombocytopenia caused by autoantibodies or direct infection of hematopoietic progenitor cells and megakaryocytes. Moreover, severe viral infection results in deficiencies in the natural anticoagulants protein C, protein S, antithrombin, and heparin cofactor II, and impaired fibrinolysis, with increased levels of plasminogen activator inhibitor-1, and antiphospholipid antibodies.^10^

### Critical illness-associated cerebral microbleeds

**Figure 6.**
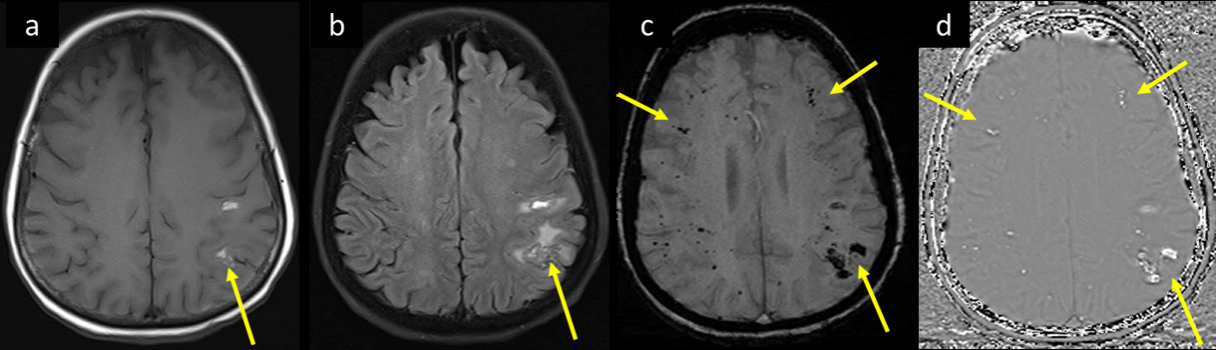
A 66-year-old female with known DM, and HTN, admitted with COVID pneumonia, progressed to severe ARDS requiring intubation, had deterioration of the level of consciousness, acute renal failure, and seizures with jerky movements of right upper limb. MRI brain axial images at supraventricular level (a) *T*_1_WI, and (b) *T*_2_-FLAIR shows pre- and post-central cortical/juxtacortical microbleeds with bright signal in *T*_1_ and FLAIR, and surrounding parenchymal edema (arrows in a, b). SWI at the same level (c) magnitude and (d) phase axial images demonstrates corresponding dephasing signal/blooming, as well as widespread punctate microhemorrhagic changes (long and short arrows respectively in c and d). ARDS, acute respiratorydistress syndrome; DM, diabetes mellitus; FLAIR, fluid attenuated inversionrecovery; HTN, hypertension; SWI, susceptibility-weighted imaging

In recently published series, extensive microbleeds were demonstrated in MRI of 12 patients during or immediately after intensive care unit (ICU) admission. The microbleeds were demonstrated only in blood sensitive T2 gradient series, diffusely involving the juxtacortical white matter and corpus callosum, but sparing the cortex, deep and periventricular white matter, basal ganglia, and thalami, with some patients also showing internal capsule or posterior fossa involvement (Figure 6).

All patients had respiratory failure, 11 out of 12 received mechanical ventilation, and 3 out of 12 received extracorporeal life support.^11^

The similarity between critical illness-associated microbleeds and high altitude exposure microbleeds suggests hypoxemia as a common factor, with hypoxia-induced hydrostatic or chemical effects on the blood–brain barrier (BBB) potentially accounting for the extravasation of erythrocytes.^12^

Moreover, another possible explanation is disseminated intravascular coagulation (DIC).^13^ In our series this was categorized in four patients by formal DIC score, and nine patients had clinical conditions ( sepsis or severe thrombocytopenia) associated with DIC.

### Hemorrhagic posterior reversible encephalopathy syndrome (PRES)

The underlying pathophysiological mechanism of increased susceptibility of COVID-19 patients to PRES remains controversial, but can be attributed to engagement of the endothelium by the virus causing damage to its lining and resulting in disruption of the BBB.^6–8^

The mechanism of microhemorrhage in PRES in COVID-19 patients can be attributed to massive release of cytokines in cytokine release syndrome resulting in damage and breakdown of the BBB, and increased coagulopathy with consumption of clotting factors as part of the DIC cascade.^14–16^

Moreover, severe pneumonia with resultant hypoxia is well-known trigger of inflammation, both at local and systemic levels.^17^

**Figure 7.**
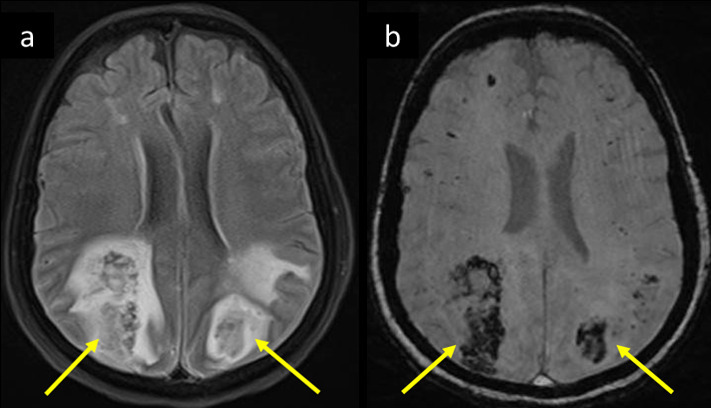
Same patient as in Figure 6 showed progressive deterioration of GCS. Her follow-up MRI 3 days later, axial (a) *T*_2_-FLAIR shows bilateral parietooccipital white matter vasogenic edema with bright signal intensity, and hemorrhagic changes showing central areas of low signal intensity consistent with hemorrhagic PRES. Corresponding blooming of hemorrhages are seen on (b) magnitude image of SWI (arrows in a and b). GCS, Glasgow coma scale;FLAIR, fluid attenuated inversion recovery; PRES, posterior reversibleencephalopathy syndrome; SWI, susceptibility-weighted imaging.

Hemorrhagic PRES (Figure 7) was noted in two of our Covid-19 PCR positive series patients with COVID pneumonia. Both of them had labile fluctuating blood pressure.

### Leukoencephalopathy with microhemorrhagic changes

Leukoencephalopathy with microhemorrhagic changes has been recently described in a COVID-19 positive 59-year-old male with known bronchial asthma, who developed progressive deterioration of respiratory symptoms, and was intubated with severe agitation and low blood pressure which required high dose analgesics and pressors.^18^

**Figure 8.**
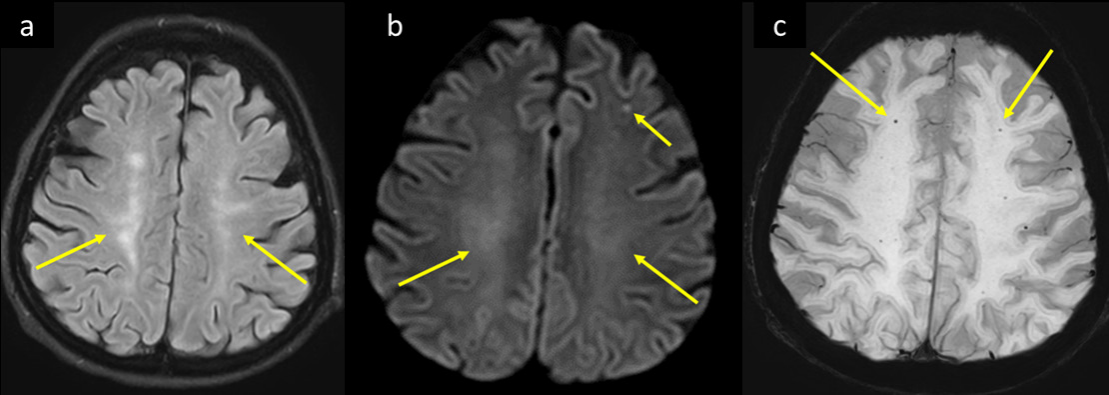
A 59-year-old female with multiple comorbidities including DM, HTN, CKD, and paroxysmal AF, presented to the ED with 2 days history of shortness of breath, fever, and myalgia. She was tested positive for COVID-19, progressed to ARDS with altered level of consciousness and developed inability to move the left side of the body. MRI brain axial image at the supraventricular level (a) *T*_2_-FLAIR shows bilateral centrum semiovale white matter hyperintensities suggesting leukoencephalopathy (arrows in a). (b) Axial DWI b1000 shows corresponding facilitated diffusion of the white matter changes and subcortical lacunar infarcts of varying ages (long and short arrows respectively in b). (c) Axial SWI shows associated scattered punctate white matter blooming microbleeds (arrows in c). ARDS, acute respiratorydistress syndrome; AF, atrial fibrillation; CKD, chronic kidney disease; DM, diabetesmellitus; DWI, diffusion-weighted image; ED, emergency department; FLAIR, fluidattenuated inversion recovery; HTN, hypertension; SWI, susceptibility-weightedimaging.

MRI of the brain in our patient with leukoencephalopathy and microhemorrhages revealed diffuse confluent posterior predominant white matter *T*_2_/FLAIR hyperintensities, and scattered microhemorrhages, without diffusion restriction (Figure 8). These imaging findings are non-specific and can accompany several well-established leukoencephalopathies, such as acute hemorrhagic encephalomyelitis.

The mechanism of white matter injury with associated microhemorrhages and its relationship to COVID-19 infection is proposedly multifactorial, including viral neurotropism with viral endothelial injury, cytokine storm cascade changes with secondary coagulopathy, and thrombotic microangiopathy.^19^

### Hemodynamic ischemic-hypoxic events

**Figure 9.**
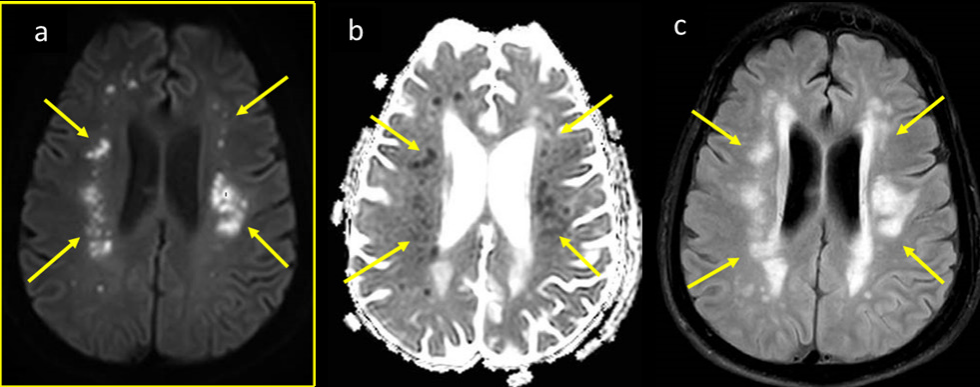
A 56-year-old male COVID-19 positive patient with severe ARDS was intubated and admitted to ICU and was not waking up subsequently. MRI brain axial images at high ventricular level (a) DWI b1000, (b) corresponding ADC map, and (c) axial *T*_2_-FLAIR, show parasagittal white matter scattered chain of nodular (rosary beaded) diffusion restriction with high signal on DWI, low signal on ADC map (arrows in a, b), with more widespread *T*_2_-FLAIR white matter bright signal vasogenic edema (arrows in c). ADC, apparent diffusion coefficient; ARDS, acute respiratory distress syndrome; DWI, diffusion-weightedimage; FLAIR, fluid attenuated inversion recovery; ICU, intensive care unit.

**Figure 10.**
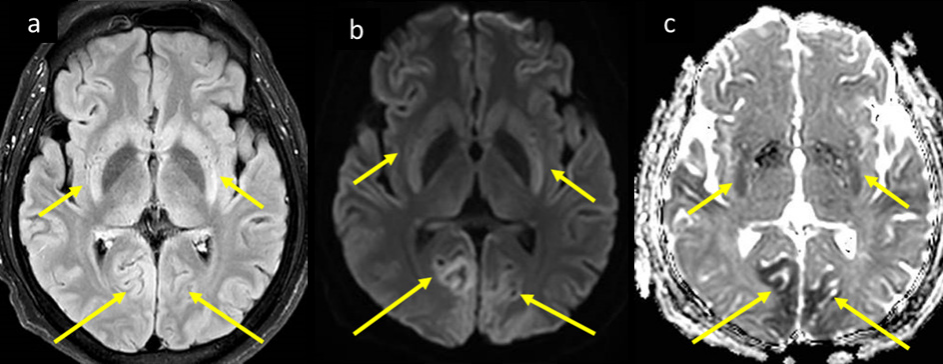
A 49-year-old COVID-19 positive male progressed to ARDS, and developed cardiac arrest for short period during endotracheal intubation. MRI brain axial images at the level of basal ganglia, (a) *T*_2_-FLAIR, (b) DWI b1000, and (c) ADC map, shows bilateral cerebral gyral and basal ganglia swelling and bright signal on *T*_2_-FLAIR, corresponding diffusion restriction with high signal on DWI signal and low signal on ADC map, consistent with hypoxic ischemic encephalopathy (long and short arrows respectively in a–c). ADC, apparent diffusion coefficient; ARDS, acute respiratory distress syndrome; DWI, diffusion-weightedimage; FLAIR, fluid attenuated inversion recovery; ICU, intensive care unit.

**Figure 11.**
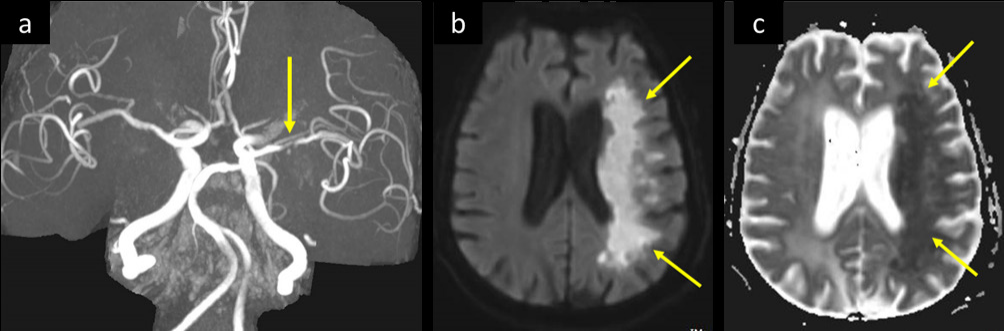
A 66-year-old male presented to ED with fatigue, cough, and fever, and was tested positive for COVID-19. He had multiple comorbidities including CAD, old cerebellar stroke, and DM. He developed pneumonia with rapid deterioration to ARDS which required intubation, and there was progressive deterioration of level of consciousness. (a) 3D-MIP- MRA shows severe stenosis of M1 segment of left MCA (arrow in a). (b) axial MRI DWI b1000 and corresponding (c) ADC map shows left-sided diffuse white matter diffusion restriction with bight signal intensity in (b) and low signal in (c), (arrows in b, c). ADC, apparent diffusion coefficient; CAD, coronary artery disease; DWI, diffusion-weighted image; ED,emergency department; DM, diabetes mellitus; MCA, middle cerebral artery; MIP, maximumintensity projection; MRA, MR angiography.

Three brain imaging hemodynamic hypoperfusion-hypoxic patterns were encountered in our COVID-19 case series, including watershed infarcts (Figure 9), hypoxic ischemic encephalopathy (Figure 10), and delayed post-hypoxic leukoencephalopathy (Figure 11).

Watershed cerebral infarctions (arterial border zone infarcts) occur at the border between cerebral vascular territories, usually related to hemodynamically significant arterial stenosis and severe hypotensive episodes. It can be gyriform in the cortical (external) border zones, or involve the deep (internal) border zones parallel to the lateral ventricles in the centrum semiovale or corona radiata.^20^ Hypoxic ischemic encephalopathy (global hypoxic ischemic injury) often follows an acute event like asphyxia, cardiac/respiratory arrest or diffuse cerebrovascular disease in adults. It predominantly affects the gray matter structures (basal ganglia, thalami, cerebral cortex) due to their high metabolic requirement.^21^ Delayed post-hypoxic leukoencephalopathy is a rare entity which can follow an acute hypoxic episode, characterized by initial neurological deterioration, followed by clinical improvement with return to baseline or near baseline, and subsequent neurological decline with a median time to relapse of around 14–30 days. The proposed pathophysiological mechanism relates to the fact that the turnover rates for some myelin-related proteins range between 19 and 22 days, which is close to the average time for clinical relapse after initial injury.^22^

### Meningoencephalitis and flare-up of other infections

**Figure 12.**
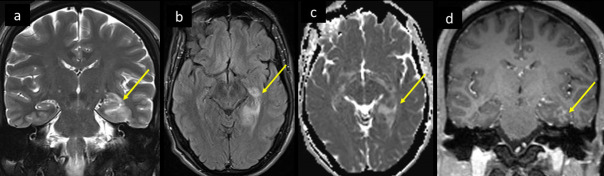
34-year-old male, presented with to the ED with 4 days of mild fever and flu like symptoms, with increasingly severe headache, photophobia, and dizziness for 2 days. He tested positive for COVID, and all his CSF work-up was negative. MRI brain coronal *T*_2_ (a) shows mildly swollen bright hippocampus and medial temporal lobe, axial FLAIR-*T*_2_ (b), ADC (c) and cororonal *T*_1_ post-i.v. contrast, shows corresponding bright signal, facilitated diffusion, and patchy post-contrast enhancememnt respectively (arrow in a–d). ADC, apparent diffusion coefficient; CSF, cerebrospinal fluid; FLAIR, fluid attenuated inversionrecovery

Two patterns of encephalitis have been recently reported in COVID-19 patients, medial temporal hippocampal involvement (Figure 12) and hemorrhagic necrotizing encephalitis patterns.

It is reported that SARS-CoV genome sequences were detected in the brain of all SARS autopsies with real-time RT-PCR, with strong signals in the hippocampus.^23^ Recent study claims that the genomic sequence of SARS-CoV and SARS-CoV-2 is similar. Especially, the receptor binding domains of SARS-CoV are structurally similar to that of SARSCoV-2.

Accordingly, SARS-CoV and SARS-CoV-2 share ACE2 as a receptor and invade the same regions in human brain.^24,25^ Moriguchi et al, recently reported COVID-19 meningoencephalitis in a COVID-19 patient with progressive deteriorating pneumonia, meningismus, multiple epileptic seizures, and deteriorating level of consciousness. CSF analysis was positive for SARSCoV-2 RNA and negative for HSV and varicella-zoster antibodies, and brain MRI showed abnormal bright *T*_2_ signal involving the hippocampus.^26^ On the other hand, Poyiadji et al^27^ reported a presumptive case of COVID19 associated acute necrotizing hemorrhagic encephalopathy, in a female airline worker with SARS-CoV-2, and CSF analysis showing negative bacterial culture and virus panel. Her brain MRI demonstrated hemorrhagic rim enhancing lesions in thalami, medial temporal lobes and subinsular regions bilaterally, and she was started on i.v. immunoglobulin.^27^

Acute necrotizing encephalopathy (infection-induced acute encephalopathy) is a rare complication triggered by viral infections including influenza. And has been found to be associated with intracranial cytokine storms, leading to Blood-Brain barrier (BBB) breakdown without neuroinvasion or parainfectious demyelination.^28^

Acute necrotizing encephalopathy has been mainly described in the pediatric population, but can also occur in adults. The most important MRI features include multifocal involvement of the brain most characteristically in the thalamus, brain stem, cerebral white matter, and cerebellum. The lesions show *T*_1_-FLAIR hyperintense signal with internal hemorrhage, and post-contrast images show ring enhancement.^29^

**Figure 13.**
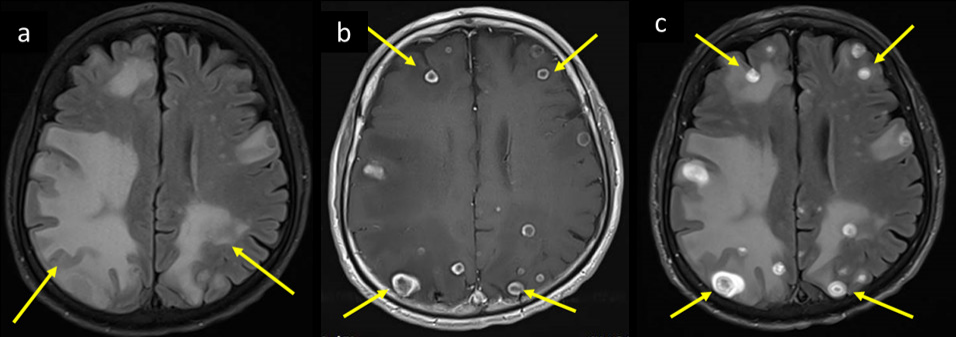
A 65-year-old male, known heavy smoker with COPD presented to the ED with fever, increasing cough, severe headache, and brief episodes of left arm stiffness, face asymmetry, and aphasia. He rapidly progressed to status epilepticus with drop of GCS. He was intubated for airway protection and shifted to MICU, and started on antiepileptic medication. His PCR COVID-19 tested positive and CSF PCR was positive for TB. Brain MRI axial images at supraventricular level (a) *T*_2_-FLAIR, (b) corresponding *T*_1_WI post-i.v. contrast and (c) repeat *T*_2_-FLAIR post-i.v. contrast shows bilateral cerebral widespread vasogenic edema (long arrows in a–c), which surround ring enhancing lesions with central low signal intensity core (short arrows in a–c) representing extensive - flare up of tuberculomas. CSF, cerebrospinal fluid;COPD, chronic obstructive pulmonary disease; ED, emergency department; FLAIR, fluidattenuated inversion recovery; GCS, Glasgow coma scale; MICU, medical intensivecare unit; PCR, polymerase chain reaction; TB, tuberculosis.

**Figure 14.**
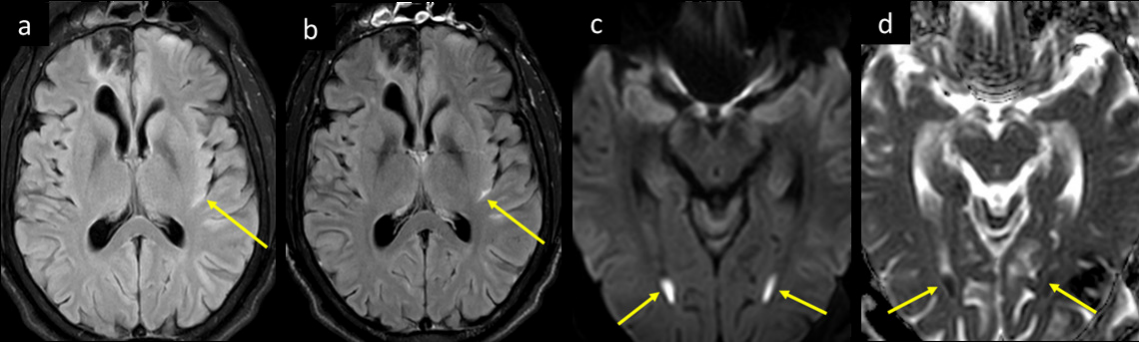
A 27-year-male patient with known post-traumatic seizures on antiepileptic mediations presented to ED with status epilepticus. He was found to be febrile and desaturated, the seizures were aborted, and he was intubated, and tested positive for COVID-19. Plain CT head (not shown) showed right anterior frontal old trauma changes and was otherwise unremarkable. His CSF analysis proved positive for Gram positive *Streptococcus pneumonia*. MRI head axial images at mid-ventricular level (a) *T*_2_-FLAIR before and (b) after i.v. contrast shows bright signal intensity in sylvian fissure, with post-contrast enhancement (long arrows in a and b), consistent with meningitis. Note the post-traumatic right anterior frontal encephalomalacia and gliotic changes (short arrow in a and b). Axial images at lower ventricular level (c) DWI b 1000 and (d) ADC map shows dependent pus with diffusion restriction within the occipital horns of the lateral ventricles bilaterally, representing ventriculitis. ADC, apparent diffusion coefficient; CSF, cerebrospinal fluid; DWI, diffusion-weighted image; ED,emergency department; FLAIR, fluid attenuated inversion recovery.

Moreover, the overwhelmed immune system in COVID-19 patients can result in flare up of other infections, the examples of which are presented in our case series such as tuberculosis (Figure 13) and pneumococcal infection (Figure 14).

### Acute disseminated encephalomyelitis (ADEM)

ADEM is an uncommon monophasic immune-mediated inflammatory demyelinating disorder that typically affects the white matter of the central nervous system, usually 1–3 weeks post-upper respiratory tract viral infection. Radiologic findings of ADEM are non-specific, but features like thalamic involvement along with multifocal white matter lesions and broken ring/horseshoe like enhancement can point to the diagnosis. The clinical features and laboratory findings can also help in differentiating it from other space occupying lesions.^30^

**Figure 15.**
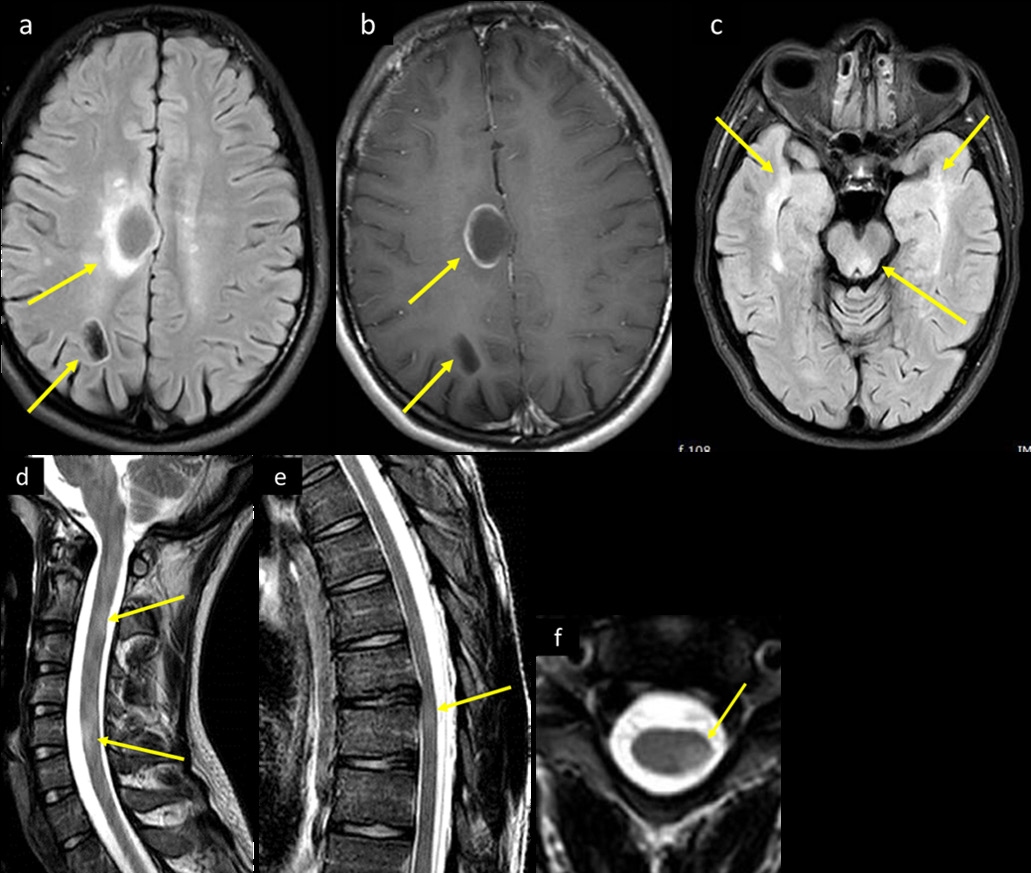
A 25-year-old male with recent history of flu-like illness before 3 weeks presented to the ED with increasing generalized body aches and upper and lower limb weakness. On examination, he had cerebellar signs and ataxia, and mildly decreased power in all 4 limbs of 4/5, with otherwise no focal neurological deficits. His COVID-19 PCR was confirmed positive. Brain CT (not shown) showed bilateral white matter cystic lesions, with surrounding mild vasogenic edema. His CSF analysis showed mildly elevated WBCs with lymphocytic predominance, mildly elevated glucose and protein. CSF culture, viral serology panel, TB PCR and autoimmune work-up were all negative. MRI brain axial (a) FLAIR image at the supraventricular level shows right frontal parasagittal and parietooccipital white matter cystic lesions, with bright intensity marginal changes. (b) Corresponding axial *T*_1_ post-i.v. contrast image shows incomplete ring enhancement of the more anterior lesion, and (c) axial FLAIR *T*_2_ image at the level of the midbrain shows posterior midbrain and bilateral anterior temporal lobes ill-defined bright signal intensity changes. MRI of the spinal cord sagittal *T*_2_ (d) cervical spine and (e) dorsal spine demonstrates multilevel short segment abnormal bright signal intensity (arrows in d and e). (f) Axial *T*_2_ image at mid-cervical level demonstrates eccentric localization of the intramedullary lesion (arrow in f). CSF, cerebrospinal fluid; ED, emergency department;FLAIR, fluid attenuated inversion recovery; PCR, polymerasechain reaction; TB, tuberculosis.

The monophasic involvement in our index case of brain and spinal cord in a known COVID positive quarantine case were consistent with ADEM (Figure 15). The mechanism of white matter injury and its relationship to COVID is uncertain, with the possibilities of post-infectious autoimmunity, as well as COVID-associated cytokine release syndrome.^31^

These mechanisms yet to be elucidated may explain the different patterns of tissue reaction resulting in cystic lesions in our index case, and the recently published case of hemorrhagic leukoencephalopathy.^32–34^

### Guillain-Barré syndrome (GBS)

**Figure 16.**
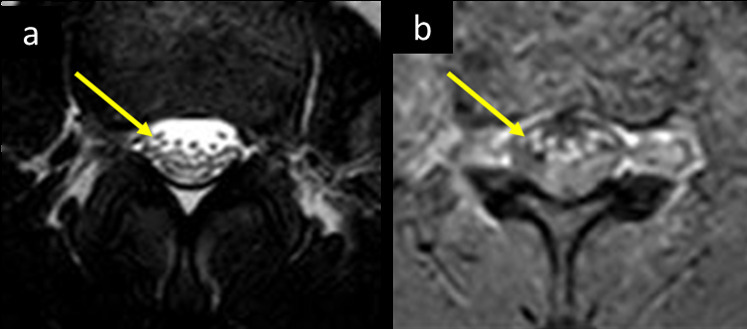
A 25-year-old male presented to the ED with upper and lower limb weakness which progressed gradually over the past 2 days, associated with pain in the upper neck region. He was also complaining of fever and diarrhea for the last 5 days, with no respiratory symptoms. He tested positive for COVID-19. His neurological examination showed power of 3/5 in all four limbs, which got worse to become 1-2/5 in all limbs in the subsequent days. He was started on IVIG with mild improvement of the lower limb weakness over next 5 weeks. MRI at upper lumbar and cauda equina level (a) axial *T*_2_ and (b) *T*_1_WI post-i.v. contrast demonstrates thickening and post contrast enhancement of intradural ventral nerve roots, consistent with Guillain-Barre syndrome (Arrows in a and b). ED, emergency department;IVIG, i.v. immunoglobulin.

GBS (acute dysimmune neuropathy), an acute sensory and motor polyradiculoneuritis has been reported with COVID-19.^35^ Alberti et al^36,37^ recently reported a 71-year-old male COVID-19 patient with moderate respiratory symptoms and paresthesia in both hands and feet, and distal weakness which rapidly evolved to a severe flaccid tetraparesis over 3 days. His CSF analysis showed a mild increase in the protein content (54 mg dl^−1^) and mild leukocytosis (9 cells µL^−1^) but was negative for SARSCoV-2. Electroneurography was consistent with a severe form of acute polyradiculoneuritis with prominent demyelinating features. Similar clinical scenario was encountered in one of our COVID-19 patients, with typical MR imaging features of GBS (Figure 16).

## Discussion

The COVID-19 pandemic in Qatar is part of the 2019–2020 worldwide pandemic with the highest number of confirmed cases per capita of any country in the world. Qatar has the second-highest total of confirmed cases in the Arab world after Saudi Arabia at 95,106 with total recoveries at 80,170, total deaths at 113, and 14,823 active cases under treatment.^1^

Out of 126 patients referred to neuroimaging, 50 cases showed neuroimaging abnormalities. The most commonly encountered neuroimaging abnormality was arterial ischemic stroke in 25 cases (30%), diffuse microhemorrhages in 13 cases (15%), cerebral venous thrombosis in 3 cases (3.6 %), hemodynamic/hypoxic ischemic changes in 4 cases (4.8%), PRES in 1 case (1.2%), meningoencephalitis exacerbation in 2 cases (2.4%), non-specific leukoencephalopathy with microhemorrhagic changes in 1 case (1.2 %), ADEM in 1 case (1.2%) and GBS in 1 case (1.2%).

In a recently published multicenter series from Italy, 51 out of 108 patients (47%) who underwent neuroimaging (in a total of 725 hospitalized patients) showed acute neuroimaging abnormalities. The neuroimaging hallmark was acute arterial ischemic strokes in 34 (31%), with 19 (18%) being large vessel infarcts, 11 (10%) small vessel infarcts, 3 (3%) cardioembolic infarcts and 1 (1%) showing hypoxic ischemic encephalopathy pattern. 6 (6%) had intracranial hemorrhages, with subarachnoid hemorrhage in 3 (3%). PRES was seen in 1 (1%), 2 (2%) had cerebral venous thrombosis, 2 (2%) multiple sclerosis plaques exacerbation, 2 (2%) GBS, 1 (1%) Miller Fisher syndrome and 2 (2%) non-specific encephalopathy with cortical pattern of *T*_2_/FLAIR hyperintense signal and associated restriction diffusion that may be attributed to systemic toxemia, viremia and/or hypoxic effects.^38^

A recently published series from Turkey reported that of the 235 patients who required ICU care, 50 patients (21%) developed neurological symptoms. Brain MRI was performed in 27 of those 50 patients with neurologic symptoms. The most common imaging finding was cortical signal abnormalities with or without subcortical white matter involvement on FLAIR images in 10/27 (37%). Associated cortical diffusion restriction, and leptomeningeal enhancement, with cortical blooming hypointensities were seen in some cases. The main differential diagnosis for this constellation of findings was encephalitis (infectious or autoimmune), hypoglycemia, hypoxia, and seizure-induced brain findings were considered in the differential diagnosis. one patient showed acute transverse sinus thrombosis and another one had acute infarction in the right middle cerebral artery territory. MR did not reveal COVID-19-related intracranial findings in 15/27 cases (56%).^39^

In another recent study from France, out of 58 COVID-19 patients admitted to the ICU neurological complications occured in 84% of the patients. Brain MRI scans were performed in 13 patients, and showed leptomeningeal enhancement in 8 of these cases.^40^

Neurological symptoms were more prevalent in patients with more severe respiratory disease.^41^ This is concordant with prior studies showing coronavirus neurotropism, attributed its affinity for the ACE2 receptor, which is a common functional receptor of both vascular endothelial cells and neurons in nervous system.^6^

A severe inflammatory reaction develops in critically ill COVID-19 patients which is hypothesized to be due to rapid T-cells and macrophages accumulation, with resultant increase in interleukin 6. This leads to multiorgan dysfunction and other clinical symptoms including fever. The extensive release of multiple cytokines into the bloodstream aiming at destroying the virus, paradoxically results in numerous clinical manifestations commonly known as cytokine release syndrome.^42,43^

The exact etiopathogenesis of neurological symptoms in COVID-19 is poorly understood. This could be due to critical illness or direct neuroinvasion of the virus. A cohort of patients with severe illness due to COVID-19 could develop cytokine storm syndrome which can act as a trigger for ischemic strokes, as a result of the prothrombotic features of the inflammatory response.^14,15^

We need more data to correlate neurotropism and other etiologies like cytokine storm syndrome, hypoxia, subclinical seizures, and critical illness-related encephalopathy with the neuroimaging findings in COVID- 19.

### Key points

1. In the ongoing COVID-19 pandemic, neurological complications have emerged as an important determinant of disease course with significant morbidity and mortality.
2. Neuroimaging plays a crucial role in the investigation of COVID-19 hospitalized patients with clinically suspected neurological complications.
3. The spectrum of neuroimaging abnormalities includes changes related to increased thrombogenicity, cytokine storm, critical illness related neurological complications, neuroinvasive nature of the virus, and post-viral encephalopathy, myelitis, and radiculopathy.
